# Ferroptosis-based therapeutic strategy targeting iron metabolism and SLC7A11 overcomes oxaliplatin resistance in patient-derived pancreatic cancer organoids

**DOI:** 10.1186/s13046-026-03745-z

**Published:** 2026-05-30

**Authors:** Jin Ho Choi, Kyung Min Lee, Minsik Oh, Janghyun Noh, Yooyeon Kim, Eunhye Hwang, In Rae Cho, Woo Hyun Paik, Ji Kon Ryu, Ja-lok Ku, Sang Hyub Lee

**Affiliations:** 1 Department of Internal Medicine Medicine and Liver Research Institute, Seoul National University Hospital, Seoul National University College of Medicine, 101 Daehak-ro, Jongno-gu, Seoul, 03080 Republic of Korea; 2 Center for Medical Innovation, Biomedical Research Institute, Seoul National University Hospital, Seoul, Republic of Korea; 3 Department of Data Technology, Myongji University, Seoul, Republic of Korea; 4 Korean Cell Line Bank, Laboratory of Cell Biology, Cancer Research Institute, Seoul National University College of Medicine, Seoul, Korea

**Keywords:** Pancreatic cancer, Ferroptosis, Organoids, Drug resistance, Biomarker

## Abstract

**Background:**

Oxaliplatin resistance remains a significant challenge in pancreatic cancer (PC) treatment. Ferroptosis, an iron-dependent form of cell death characterized by lipid peroxidation, has emerged as a promising therapeutic target for overcoming chemotherapy resistance. This study investigated whether ferroptosis induction could overcome oxaliplatin resistance in PC.

**Methods:**

We established 42 patient-derived pancreatic cancer organoids (PDPCOs) and performed comprehensive molecular profiling including whole-exome sequencing, RNA sequencing and drug response assays. Cell viability, lipid peroxidation, iron levels, mitochondrial function, and drug synergy were evaluated. Survival analysis and differential gene expression analyses were conducted according to the biomarker candidate.

**Results:**

Transcriptomic analysis revealed distinct ferroptosis-related alterations in OXA-resistant PDPCOs, including a negative enrichment of ferroptosis suppressor genes. The ferroptosis inducer artesunate (ART) synergistically enhanced anticancer effects of OXA in cell lines and PDPCOs. Mechanistic studies demonstrated that combination therapy induced ferroptotic cell death by promoting lipid peroxidation, intracellular iron accumulation, and mitochondrial dysfunction. Combination therapy remarkably inhibited tumor growth in PDPCO-derived xenograft models. ART/OXA treatment upregulated expressions of iron transport proteins transferrin receptor (TFRC) and divalent metal transporter 1 (DMT1), contributing to ferroptosis induction. Overexpression of solute carrier family 7 member 11 (SLC7A11) conferred dual resistance to OXA and ART by suppressing ferroptosis, whereas its knockdown or pharmacological inhibition with erastin sensitized resistant cells to combination therapy. Notably, triple combination of ART, OXA, and erastin effectively overcame resistance in dual-resistant PDPCOs.

**Conclusions:**

Ferroptosis-based therapeutic strategies can overcome oxaliplatin resistance in PC, supporting further investigation of SLC7A11 as a candidate biomarker for ferroptosis-based therapeutic responsiveness.

**Supplementary Information:**

The online version contains supplementary material available at 10.1186/s13046-026-03745-z.

## Introduction

Pancreatic cancer (PC) is a highly aggressive malignancy, with a 5-year overall survival rate of only 10%, and is projected to become the second leading cause of cancer-related mortality by 2030 [1, 2]. While combination chemotherapy regimens have resulted in improved survival outcomes, drug resistance and nonresponsiveness remain major obstacles to effective treatment [3, 4]. Oxaliplatin (OXA), a commonly used component of combination chemotherapy regimens in PC, is frequently limited by both intrinsic and acquired resistance, which substantially reduces its therapeutic efficacy [3, 5]. Despite extensive research, the mechanisms underlying OXA resistance remain poorly understood. As such, a critical need exists for development of novel therapeutic strategies capable of overcoming chemotherapy resistance in PC.

Ferroptosis—iron-dependent, nonapoptotic cell death characterized by lipid peroxidation and mitochondrial dysfunction—has emerged as a promising approach for targeting chemotherapy-resistant cancer cells [6–8]. Cancer cells exhibit increased iron demand compared to normal cells, rendering them susceptible to iron-catalyzed ferroptotic cell death [9]. Evidence suggests that disruptions in cell death pathways, especially ferroptosis, may contribute to chemoresistance in various cancer [10–13]. However, the potential association between OXA resistance and ferroptosis in PC remains unclear. While ferroptosis induction has shown promise in several malignancies, its therapeutic relevance in PC and its interaction with platinum-based chemotherapy remain poorly defined. Ferroptosis inducers show potential in overcoming chemotherapy resistance when used in combination with cytotoxic agents, with encouraging preclinical results [14–20]. Among these, artesunate (ART) demonstrates potent cytotoxic effects against multiple cancers [21, 22]. A prior study reported that ART induces ferroptosis in PC cells with KRAS activation, underscoring its potential [23]. Additionally, ferroptosis-related gene signatures associate with prognosis and therapeutic response in PC, suggesting a central role for ferroptosis in PC progression and treatment [24–26]. 

Cellular antioxidant defense mechanisms are essential for protecting against ferroptosis and may also contribute to chemotherapy resistance. While ferroptosis offers a targeted means of eliminating malignant cells, the mechanisms behind its synergy with conventional chemotherapeutics remain incompletely understood [27]. Elucidating how these systems intersect with drug resistance pathways could uncover new therapeutic opportunities.

This study aims to evaluate the effects of ferroptosis induction in OXA-resistant PC cells, investigate the underlying mechanisms of its interaction with chemotherapy, and identify potential biomarkers predictive of ferroptosis sensitivity. Using patient-derived pancreatic cancer organoids (PDPCOs), we sought to establish optimal conditions for inducing ferroptosis in OXA-resistant cases and propose effective strategies for overcoming OXA resistance.

## Materials and methods

### Patient-Derived Pancreatic Cancer Organoid (PDPCO) culture

The collection of PC patient data and tissue for organoid generation was performed following guidelines under the approval of the Institutional Review Board of Seoul National University Hospital. PDPCOs were established from core biopsy tissue obtained through endoscopic ultrasound-guided fine needle biopsy. All organoids were grown in medium consisting of Wnt3a/R-spondin1/Noggin-conditioned medium (50% vol/vol), containing 1× B27 supplement (Life Technologies, Carlsbad, CA, USA), 0.5 mM N-acetyl-L-cysteine (Sigma-Aldrich), 10 mM nicotinamide (Sigma-Aldrich), 50 ng/mL human epithelial growth factor (EGF; PeproTech Inc., Cranbury, NJ, USA), 500 nM A83-01, 100 ng/mL human fibroblast growth factor 10 (FGF10; PeproTech), and 10 nM gastrin (R&D Systems, Inc.; Minneapolis, MN, USA) in Advanced DMEM/F12 (Life Technologies) medium. For passaging, organoids were collected, washed, and disrupted by mechanical shearing or digestion with TrypLE Express (Life Technologies), and organoid fragments were replated in fresh Matrigel (Corning, NY, USA). All established PDPCOs were included in the analyses, no exclusions were made.

### Processing of whole-exome, whole-genome, and RNA sequencing data

Whole-exome and whole-genome sequencing data were processed using the nf-core/sarek workflow (v3.4.2) [28], following GATK Best Practices. Sequencing reads were aligned to the GRCh38 reference genome. Somatic variant calling was performed using GATK Mutect2 [29]. Variant annotation was performed using SnpEff and ANNOVAR with GRCh38 reference [30, 31]. 

RNA sequencing data were processed using the nf-core/rnaseq pipeline (version 3.14.0) [32], following RNA-seq best practices. Raw sequencing reads were first trimmed for adapter sequences and low-quality bases using fastp [33]. Trimmed reads were aligned to the GRCh38 reference genome using the STAR aligner [34]. Transcript quantification was performed using Salmon in alignment-based mode, generating gene expression data [35]. 

To identify gene expression signatures associated with drug response, PDPCOs were stratified into sensitive and resistant groups based on AUC values derived from drug response assays. Gene expression matrices were normalized and batch-corrected using pyComBat to minimize technical variability across samples [36]. Differential expression analysis was performed using the DESeq2 package [37]. Genes with a *p*-value < 0.05 and an absolute log₂ fold change ≥ 1 were considered significantly differentially expressed. To explore the biological relevance of differentially expressed genes, functional enrichment analysis was conducted using the GSEApy Python package.

### Public dataset analysis

Survival analyses were performed using the TCGA pancreatic adenocarcinoma (PAAD) cohort obtained from the Broad Institute Firehose pipeline (http://gdac.broadinstitute.org/*)* [38]. Patients were stratified by SLC7A11 expression, and progression-free and overall survival were compared by Kaplan-Meier analysis with the log-rank test. SLC7A11 expression in tumor versus non-tumor tissues was validated using GEO datasets GSE28735, GSE62452, and GSE71729.

### Cell viability assay and drug synergy assays

AsPC-1, PANC-1, and MIA PaCa-2 were selected as representative KRAS-mutant PC cell lines that exhibit heterogeneous responses to standard chemotherapeutic agents, providing a suitable panel to evaluate OXA–ART combinatorial treatment across different cellular contexts. Cell lines were cultured at 37 °C in 5% CO2. For single-agent cytotoxicity and IC50 determination, cells (3,000/well) were treated with ART (0–50 µM) or OXA (0–100 µM) for 48 h. For drug combination synergy analyses in cell lines, dose–response matrices of OXA (0–100 µM) and ART (0–20 µM) were applied for 48 h. For PDPCO synergy analyses, organoids were treated with dose–response matrices of OXA (0–500 µM) and ART (0–50 µM) for 5 days, reflecting the higher concentrations required for sufficient drug penetration in 3D culture. For inhibitor assays, cells were preincubated with Fer-1 (1 µM, 2 h), Trolox (40 µM, 6 h), DFO (AsPC-1 and Panc-1, 1 µM, 6 h; MiaPaCa-2, 6 µM, 6 h), and z-VAD.fmk (10 µM, 2 h) before treatment with ART. For fixed-dose combination treatments used in downstream mechanistic assays, cells were treated with OXA (AsPC-1 and Panc-1, 100 µM; MiaPaCa-2, 25 µM) and ART (AsPC-1, 10 µM; Panc-1 and MiaPaCa-2, 20 µM); treatment durations varied by assay as indicated in the corresponding figure legends. Cell viability was assessed using Cell Titer-Glo luminescent cell viability assay kit (Promega, Madison, USA).

For PDPCOs, 600 viable cells were seeded per well in 50 µL (50% Matrigel : 50% human complete organoid media). After 72 h treatment, 3D cell viability was assessed using Cell Titer-Glo 3D luminescent cell viability assay kit (Promega). Luminescence was measured on a Luminometer (Glomax^®^Explore Multimode Microplate Reader, Promega, USA). Data were normalized to vehicle control and IC50 values were calculated using Hill’s equation for GraphPad Prism software 10 (GraphPad Inc., San Diego, CA, USA).

Drug combination effects were analyzed using SynergyFinder (version 2.0) that implemented four reference synergy models (highest single agent, Bliss, Loewe, and zero interaction potency) [39]. According to the synergy score, the interaction between two drugs is likely to be antagonistic (less than − 10), additive (from − 10 to 10), and synergistic (larger than 10).

### Ferroptosis mechanism validation

Lipid peroxidation was assessed using C11-BODIPY dye (Thermo Fisher Scientific, 5 µM, 30 min) by flow cytometry. Intracellular iron levels were measured using Calcein-AM assay Kit (Thermo Fisher, 1 µM, 15 min). Mitochondrial and cellular ROS were detected using MitoSOX red (Invitrogen, 5 µM, 15 min) and H2DCFDA (Invitrogen, 5 µM, 20 min) respectively. For ferroptosis validation, cells were pre-incubated with ferrostatin-1 (Fer-1, 1 µM, 2 h), deferoxamine (DFO, 1–6 µM, 6 h), or Trolox (40 µM, 6 h) before treatment. Western blotting was performed using antibodies against SLC7A11, SLC3A2, SLC11A2 (DMT1), NCOA4, FTH1, NRF2, IRP1, IRP2 (Cell Signaling Technology, Danvers, MA, USA), TFRC (Thermo Fisher), Lamin B1, α-tubulin, and β-actin or GAPDH (BD Biosciences) as loading controls. For qPCR analysis, total RNA was isolated, cDNA was synthesized, and data were normalized to GAPDH expression levels as described in Supplementary Methods.

### SLC7A11 functional studies

SLC7A11, TFRC, DMT1, and NRF2 were silenced by siRNA-mediated knockdown (10 or 20 nM, 48 h). For gain-of-function studies, SLC7A11-overexpressing (SLC7A11-OE) Panc-1 and MIA PaCa-2 stable cell lines were generated by lentiviral transduction, with empty vector–transduced cells as controls. For pharmacological SLC7A11 inhibition, erastin (0–50 µM) or sulfasalazine (0–1,000 µM) was used in triple combination with OXA (0–500 µM) and ART (0–50 µM) in PDPCOs for 5 days. NRF2 transcriptional activity was assessed by ARE-luciferase reporter assay, and intracellular GSH levels were measured using the GSH-Glo assay. Detailed procedures are described in Supplementary Methods.

### Patient-derived organoid xenograft model

All animal studies were approved by the institutional animal care and use committee of Seoul National University Hospital (IACUC No. 21-0249-S1A0). Five-week-old female BALB/c nude mice were purchased from Orient Bio Inc. (Seongnam, Korea). Mice were acclimatized for one week before experiments. PDPCO SNU-4425-TO (5 × 10^6 cells) were suspended in 100 µL of PBS (1:1) and subcutaneously injected into the right flank of the mice. When tumors reached approximately 100 mm^3, mice were randomly divided into four groups (*n* = 5 per group): vehicle control, ART (20 mg/kg, i.p., daily), OXA (5 mg/kg, i.p., twice weekly), and combination. Tumor volume was measured twice weekly using digital calipers and calculated as (length × width^2)/2. After 21 days of treatment, mice were sacrificed, and tumors were excised, weighed, and processed for further analysis. All xenografted mice were included. Treatments were administered unblinded, but tumor measurement and analysis were performed blinded.

### Statistical analysis

Data represent mean ± SEM and were analyzed using Student’s t-test or one-way ANOVA, as appropriate. Tumor growth curves were analyzed using two-way ANOVA. For differential gene expression (DEG) analysis, genes with an absolute log2 fold change ≥ 1 and *P* < 0.05 were considered significantly differentially expressed. For survival analysis, patients were stratified into high and low expression groups based on the median biomarker candidate gene expression levels. Survival rates at 6 and 12 months were compared between high and low expression groups in both the overall cohort and oxaliplatin-treated cohort. Additionally, biomarker candidate gene expression levels were compared between survivors and non-survivors at 6 and 12 months. Kaplan-Meier survival curves were generated using the *survival* and *survminer* packages in R (Version 4.4.0), with comparisons performed using log-rank tests. Statistical analyses and data processing were performed using Prism software 10 (GraphPad Inc.) and R version 4.4.0. A difference was considered statistically significant at the *P* < 0.05. No a priori sample size calculation was performed, and sample sizes were determined based on feasibility.

### Ethics approval

This study was approved by the Institutional Review Board of Seoul National University Hospital, Korea (IRB No. 2201-138-1294; IRB no. 1102-098-357; 1712-056-905; 2003-189-1112). The animal experiments were approved by the Institutional Animal Care and Use Committee of Seoul National University Hospital (IACUC No. 21-0249-S1A0). Written informed consent was obtained from all patients for tissue collection, organoid generation, and molecular profiling. All procedures involving human participants and animal experiments were conducted in accordance with relevant ethical guidelines and regulations.

### Patient and public involvement

This study did not involve patients or the public in its design, conduct, reporting, or dissemination plans as it was a preclinical, laboratory-based study.

## Results

### Baseline molecular features and oxaliplatin responsiveness in PDPCOs

**Fig. 1:**
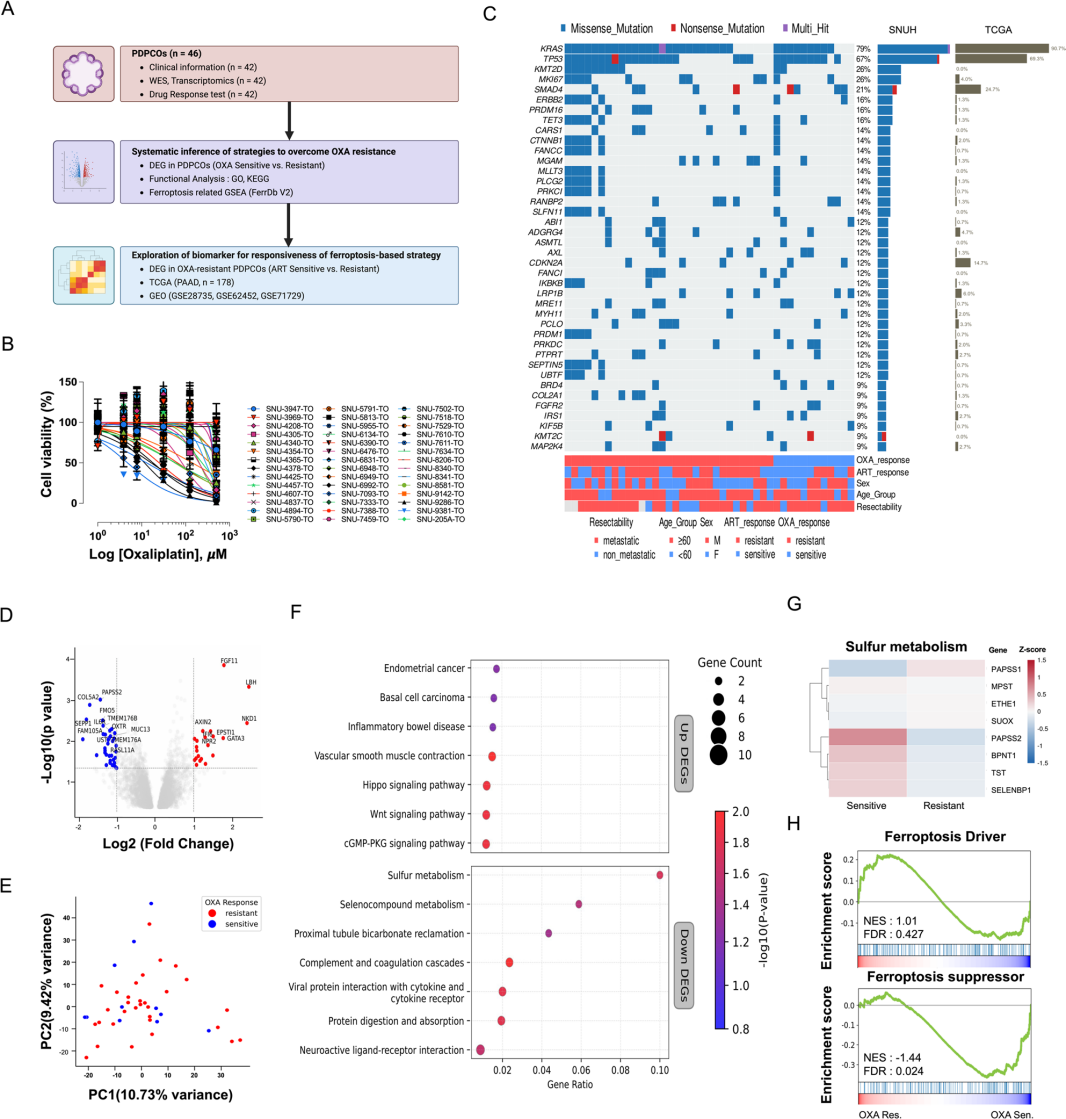
Molecular profiling of patient-derived pancreatic cancer organoids (PDPCOs) reveals ferroptosis-related mechanisms associated with oxaliplatin responsiveness. **A** Schematic illustration of the experimental workflow for establishing PDPCO (*n* = 42), performing integrative molecular profiling, and characterizing oxaliplatin resistance. **B** Dose–response curves showing the heterogeneous response to oxaliplatin (0–500 µM; 5 days) in 42 PDPCOs, measured using the 3D CellTiter-Glo assay. **C** Oncoplot displaying recurrent genomic alterations in PDPCOs compared with TCGA pancreatic cancer data. Clinical annotations, including oxaliplatin response, histology, and patient metadata, are given below. **D** Volcano plot of differentially expressed genes between OXA-sensitive and OXA-resistant PDPCOs. Red and blue dots represent upregulated and downregulated genes, respectively (log2 fold change > 1 or < − 1, *p* < 0.05). **E** Principal component analysis (PCA) reveals a distributional shift with substantial overlap between OXA-resistant (red) and OXA-sensitive (blue) PDPCOs. **F** Pathway enrichment analysis identifies enriched biological pathways differing between OXA-sensitive and OXA-resistant groups (dot size represents gene count; color intensity indicates statistical significance). **G** Heatmap showing differential expression of sulfur metabolism-related genes between OXA-resistant and OXA-sensitive PDPCOs. **H** Gene set enrichment analysis (GSEA) of ferroptosis driver and suppressor gene sets from FerrDb V2, showing significant enrichment of ferroptosis suppressor genes in OXA-sensitive, whereas ferroptosis driver genes showed no significant enrichment

We established a panel of 42 PDPCOs and collected comprehensive data—including clinical information, whole-exome sequencing, transcriptomics, and drug response profiles—for 42 of them (Fig. 1a). The patients’ clinical characteristics, including histological subtypes, age, sex, and disease status, are summarized in Table S1. This panel represents a diverse spectrum of patient demographics and disease stages, providing a clinically relevant platform for evaluating therapeutic responses in PC.

Cell viability assays revealed substantial heterogeneity in OXA responses across the 42 PDPCOs (Fig. 1b). Based on area under the curve and drug response profiles, 73.81% (31 of 42) of the organoids were classified as OXA-resistant and 26.19% (11 of 42) as OXA-sensitive.

Genetic profiling revealed mutation patterns consistent with established PC genomic signatures (Fig. 1c). The most frequent alterations were observed in KRAS (79%), followed by TP53 (67%), SMAD4 (21%), and CDKN2A (12%). These mutation frequencies align with the canonical genomic landscape of PC [40, 41] and are consistent with data from The Cancer Genome Atlas (TCGA).

### Ferroptosis-related transcriptomic signatures define oxaliplatin resistance in PDPCOs

To identify molecular correlates of OXA resistance, we examined transcriptomic differences between OXA-resistant and OXA-sensitive PDPCOs. Differential gene expression analysis revealed distinct transcriptomic profiles between OXA-sensitive and OXA-resistant PDPCOs (Fig. 1d). Principal component analysis (PCA) revealed a distributional shift between OXA-resistant (red dots) and OXA-sensitive (blue dots) organoids along PC1 and PC2, while showing substantial overlap, suggesting underlying transcriptomic differences (Fig. 1e). Pathway enrichment analysis identified several differentially enriched biological pathways between the two groups (Fig. 1f), notably those related to sulfur and selenocompound metabolism, previously implicated in redox balance and cell death regulation. Additional enrichment was observed in signaling pathways such as Hippo, Wnt, and cGMP-PKG, suggesting that diverse molecular processes may influence oxaliplatin responsiveness.

Among sulfur metabolism-related genes, PAPSS2 was relatively enriched in OXA-sensitive PDPCOs and reduced in OXA-resistant PDPCOs, whereas other genes showed variable or modest differences (Fig. 1g). Gene set enrichment analysis (GSEA) of ferroptosis-related gene signatures using the FerrDb v2 database [42] revealed that ferroptosis suppressor genes were significantly enriched in OXA-sensitive PDPCOs (NES = − 1.44; FDR q-value = 0.024), whereas ferroptosis driver genes showed no significant enrichment (NES = 1.01; FDR q-value = 0.427; Fig. 1h). These results suggest that downregulation of ferroptosis suppressor gene expressions may contribute to OXA resistance. Additional gene ontology analyses, including selenocompound and glutathione metabolism pathways, are presented in Sup. Figure 1a and Sup. Figure 1b, respectively.

Overall, these findings highlight a potential association between ferroptosis regulation and oxaliplatin resistance, supporting the rationale for combining ferroptosis inducers with conventional chemotherapy in PC therapy. The negative enrichment of ferroptosis suppressor genes in OXA-resistant PDPCOs suggests its application in potential therapeutic opportunities through ferroptosis modulation. Based on this hypothesis, we evaluated the efficacy of the combination of oxaliplatin with ferroptosis inducers in PC models.

### Combination of oxaliplatin and artesunate synergistically induces ferroptotic cell death

To determine whether ferroptosis induction could overcome OXA resistance, we evaluated the synergistic effects of ferroptosis inducers in OXA-resistant PDPCOs. Among the tested agents—ART, sorafenib, and sulfasalazine—ART showed the most potent ferroptosis-inducing activity. Its cytotoxic effects were reversed by ferroptosis inhibitors (ferrostatin-1, DFO, and Trolox), but not by the apoptosis inhibitor zVAD.fmk (Sup. Figure 2a, 2b). ART was thus selected for combination studies with OXA due to its specificity and favorable safety profile.

**Fig. 2:**
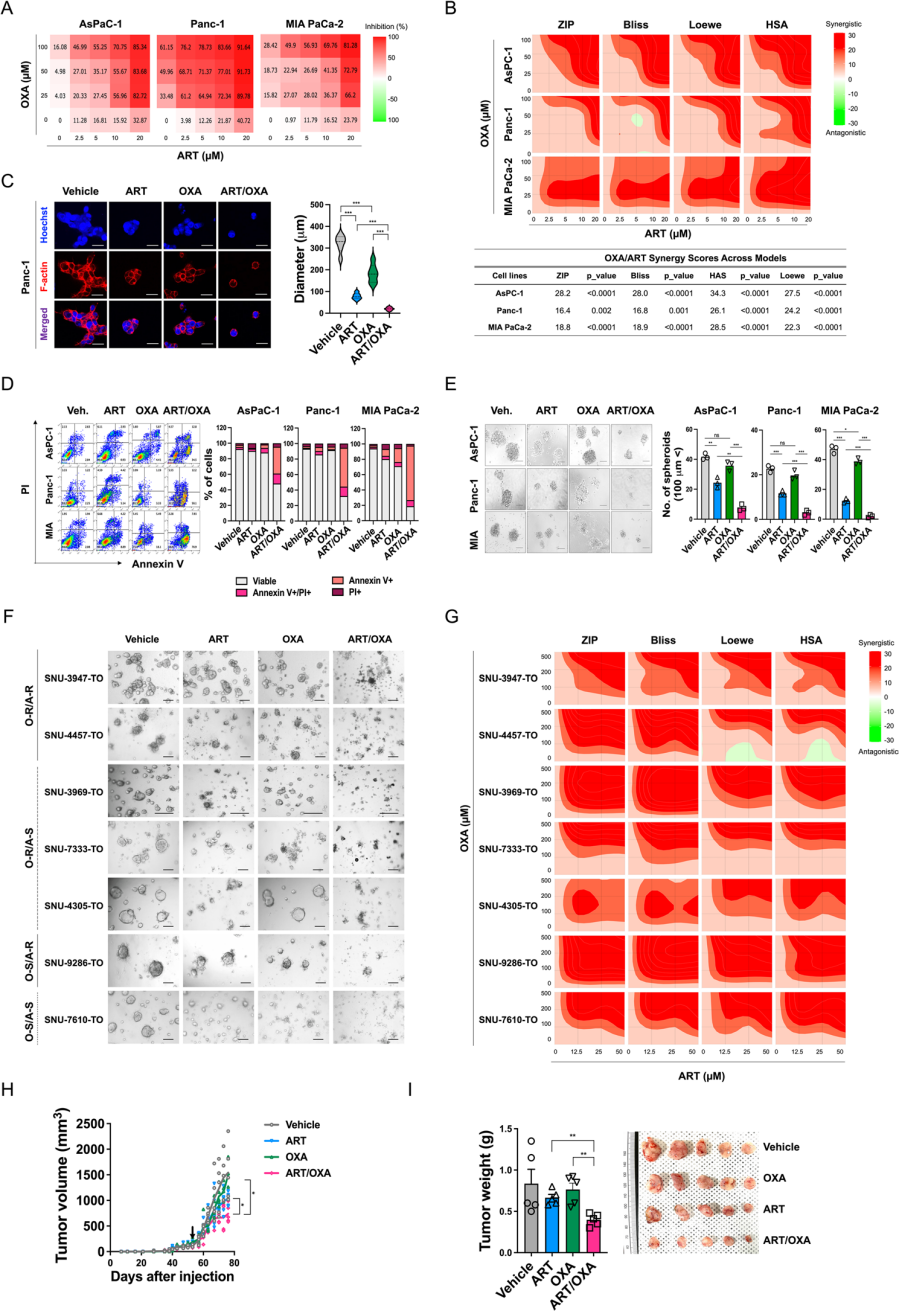
Oxaliplatin and artesunate synergize to suppress cancerous effects pancreatic cancer growth in vitro and in vivo. **A** Cell viability matrices showing the dose-dependent effects of OXA (0–100 µM) and ART (0–20 µM) combinations on AsPC-1, Panc-1, and MIA PaCa-2 cells over 48 h. The color scale indicates percent inhibition. **B** Synergy maps for OXA/ART combinations generated using ZIP, Bliss, Loewe, and HSA scoring, with corresponding synergy and statistical significance scores. **C** Representative images and quantification of cancer cell metastasis in 3D Matrigel models treated with vehicle, OXA (100 µM), ART (10 µM), or their combination for 48 h (nuclei: blue; F-actin: red). **D** Annexin V/PI flow cytometry shows cell death profiles following 48-h treatment with vehicle, OXA (100 µM), ART (10 µM), or combination. Representative flow cytometry plots and quantification of viable, early apoptotic, and late apoptotic/necrotic populations. **E** Spheroid formation capacity in 3D culture after 5-day treatment with vehicle, OXA (50 µM), ART (5 µM), or combination, with representative phase-contrast images and quantification. **F** Representative phase-contrast images of PDPCOs treated with the vehicle, ART (10 µM), OXA (200 µM), or combination for 5 days. **G** Synergy maps for OXA (0–500 µM) and ART (0–50 µM) combinations treated for 5 days across multiple PDPCOs analyzed using SynergyFinder 2.0. Four different synergy scoring models (ZIP, Bliss, Loewe, and HSA) were applied to quantify drug interactions. The color scale indicates synergistic (red) to antagonistic (green) effects. **H** Tumor growth curves of SNU-4425-TO xenografts treated with vehicle, ART (20 mg/kg, i.p., daily), OXA (5 mg/kg, i.p., twice weekly), or combination (*n* = 5 mice/group). Individual mouse data points are shown with mean values connected by lines. **I** Final tumor weight and representative images of excised tumors from each treatment group. Quantitative data are presented as mean ± SEM with individual data points overlaid. Statistical analyses were performed using Student’s t-test for two-group comparisons, one-way ANOVA followed by Tukey’s post hoc test for multi-group comparisons, and two-way ANOVA for tumor growth curve analysis. *p < 0.05, **p < 0.01, ***p < 0.001

To evaluate whether ART could enhance OXA sensitivity across diverse cellular contexts, we examined three widely used KRAS-mutant PC cell lines—AsPC-1, Panc-1, and MIA PaCa-2—that exhibit heterogeneous responses to standard chemotherapy. The OXA/ART combination therapy significantly reduced cell viability across the three PC cell lines (Fig. 2a). Synergy analyses revealed strong synergistic interactions: AsPC-1 (ZIP = 28.2; *p* < 0.0001), Panc-1 (ZIP = 16.4; *p* = 0.002), and MIA PaCa-2 (ZIP = 18.8, *p* < 0.0001) (Fig. 2b). Additional synergy metrics confirmed these findings. The combination therapy also contributed to a suppression in cancer cell metastasis in a 3D Matrigel model (Fig. 2c), reduced invasion (Sup. Figure 3), increased cell death (Fig. 2d), and impaired spheroid formation (Fig. 2e), indicating inhibition of metastasis and cancer cell stemness.

The clinical relevance of this combination therapy was further validated using our characterized PDPCO panel, previously profiled for OXA and ART sensitivity (Fig. 1b, Sup. Figure 4, Sup. Table 1). Among seven representative PDPCOs with diverse sensitivity profiles (Fig. 2f), the treatment consistently reduced growth and showed synergistic effects (Fig. 2g), with notable high synergy scores in SNU-9286-TO (ZIP = 56.0; *p* < 0.0001) and SNU-4457-TO (ZIP = 44.9; *p* < 0.0001). All tested PDPCOs showed strong synergy across multiple metrics (Sup. Table 2).

In a xenograft model using the dual-resistant PDPCO SNU-4425-TO, the OXA/ART combination promoted greater tumor suppression than either of the monotherapy, as measured by tumor volume (Fig. 2h) and final tumor weight (Fig. 2i; *p* < 0.01). This suggests that the OXA/ART combination can overcome resistance mechanisms that limit the efficacy of each agent individually.

### Iron transporters TFRC and DMT1 drive ferroptotic synergy of oxaliplatin and artesunate

**Fig. 3:**
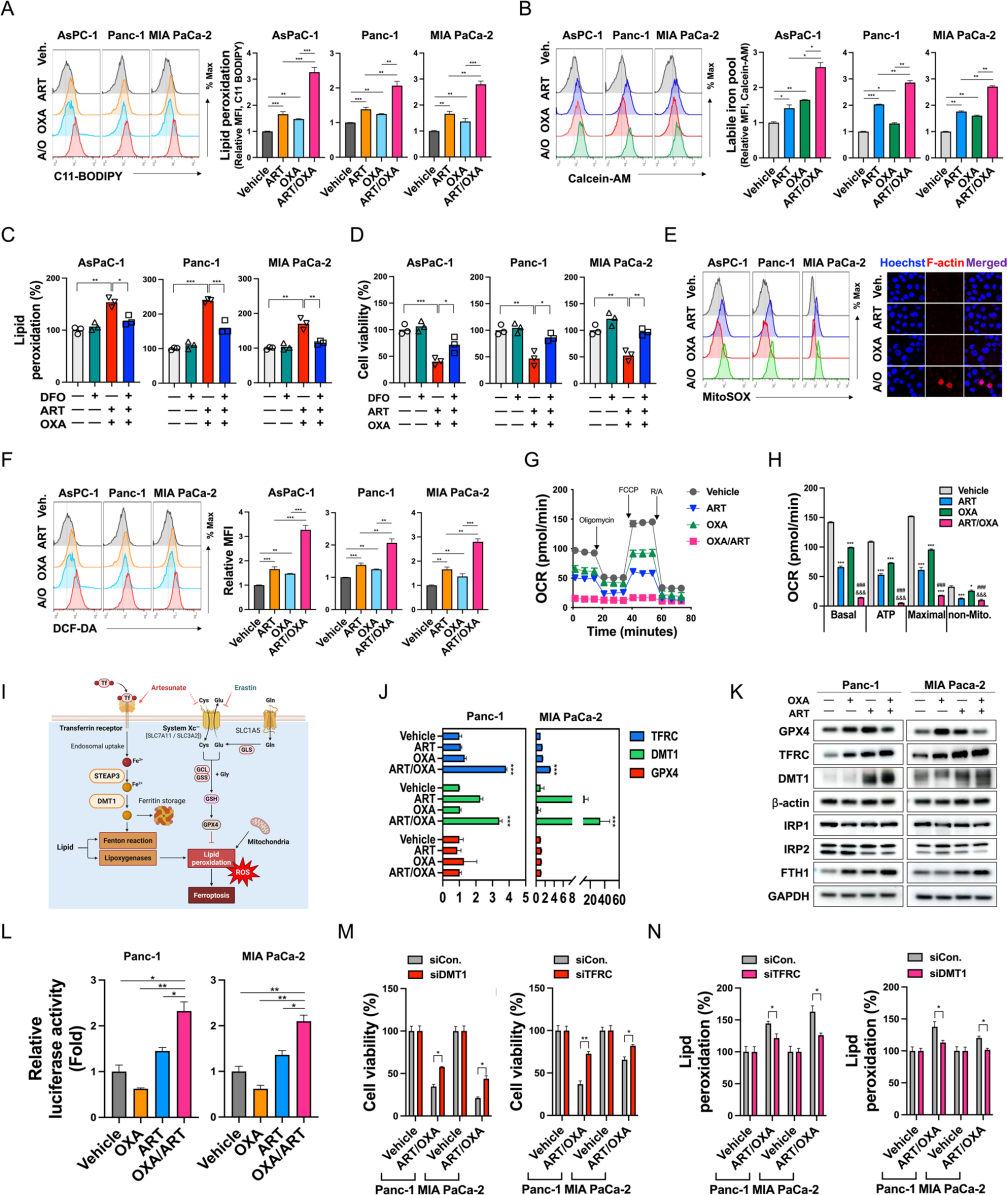
TFRC and DMT1 are essential mediators of ART-/OXA-induced ferroptosis in pancreatic cells. **A** Flow cytometric analysis of lipid peroxidation using C11-BODIPY (2 µM, 30 min) in AsPC-1, Panc-1, and MIA PaCa-2 cells treated with vehicle, ART (20 µM), OXA (200 µM), or ART/OXA combination for 24 h. Representative histograms (left) and quantification (right) are shown. **B** Calcein-AM Red (1 µM, 15 min) assay showing labile iron pool levels in PC cells treated with the indicated compounds for 24 h. Higher fluorescence indicates higher iron levels. **C** Lipid peroxidation in PC cells treated with vehicle, DFO (100 µM), ART (20 µM), OXA (200 µM), or their combinations for 24 h, quantified as a percentage of control via C11-BODIPY staining and flow cytometry. **D** Cell viability after 48 h of treatment with the indicated compounds, assessed using the CellTiter-Glo assay. **E** MitoSOX (5 µM, 15 min) staining showing mitochondrial ROS levels in PC cells treated with vehicle, ART (20 µM), OXA (200 µM), or ART/OXA combination for 24 h. Representative histograms (left) and immunofluorescence images (right) are shown. **F** DCF-DA (5 µM, 20 min) staining showing cellular ROS levels in pancreatic cancer cells treated with the indicated compounds for 24 h. Representative histograms (left) and quantification (right) are shown. **G** Oxygen consumption rate (OCR) measured in real-time using the Seahorse XF analyzer in pancreatic cancer cells treated with vehicle, ART (20 µM), OXA (200 µM), or ART/OXA combination for 24 h. Additions of oligomycin (1 µM), FCCP (1 µM), and rotenone/antimycin A (0.5 µM each) are indicated. **H** Basal, ATP-linked, maximal, and nonmitochondrial OCR values calculated from (**G**). **I** Schematic illustration of iron metabolism and the ferroptosis pathway showing transferrin receptor, System Xc-, DMT1, ferritin storage, Fenton reaction, lipid peroxidation, and ROS generation. **J** mRNA expression of TFRC, DMT1, and GPX4 in Panc-1 and MIA PaCa-2 cells treated with vehicle, ART (20 µM), OXA (200 µM), or ART/OXA for 24 h, determined by qRT-PCR and normalized to GAPDH. **K** Western blot analysis of GPX4, TFRC, DMT1, β-actin, IRP1, IRP2, FTH1, and GAPDH in Panc-1 and MIA PaCa-2 cells after 48 h of treatment with the indicated conditions. **L** NRF2 transcriptional activity measured by an ARE-luciferase reporter assay in Panc-1 and MIA PaCa-2 cells following treatment with vehicle, OXA (40 µM), ART (5 µM), or ART/OXA combination for 24 h. Data are presented as fold change in firefly/Renilla luciferase activity relative to vehicle. **M** Cell viability in Panc-1 and MIA PaCa-2 cells 48 h after transfection with siControl, siTFRC, or siDMT1 (20 nM), treated with vehicle or ART (20 µM)/OXA (200 µM) for 24 h, assessed by CellTiter-Glo. **N** Lipid peroxidation in siControl-, siTFRC-, or siDMT1-transfected Panc-1 and MIA PaCa-2 cells treated with vehicle or ART/OXA for 24 h, assessed by C11-BODIPY and flow cytometry. Unless otherwise stated, all quantitative data are presented as mean ± SEM from at least three independent experiments and were analyzed using Student’s t-test for pairwise comparisons. Statistical comparisons are denoted as follows: *p < 0.05, **p < 0.01, ***p < 0.001 vs. vehicle control; #*p* < 0.05, ##*p* < 0.01, ###*p* < 0.001 vs. OXA alone; &*p* < 0.05, &&*p* < 0.01, &&&*p* < 0.001 vs. ART alone

To delineate which axis of ferroptosis regulation primarily mediates the synergistic effect of ART and OXA, we examined the key factors involved in ferroptosis pathways. The ART/OXA combination significantly increased lipid peroxidation in all three PC cell lines, as measured by C11-BODIPY staining (Fig. 3a, *p* < 0.001), by more than twofold, as observed with single-agent treatments (*p* < 0.01), indicating the potent activation of ferroptotic pathways. An increase in intracellular iron levels as measured by Calcein-AM staining, was observed across all tested cell lines (Fig. 3b, *p* < 0.01), and lipid peroxidation (Fig. 3c) and cell death (Fig. 3d) were reversed by the activity of the iron chelator DFO (*p* < 0.001), thus establishing ferroptosis as the primary mechanism.

Furthermore, MitoSOX and DCF-DA staining revealed elevated concentrations of mitochondrial and cellular reactive oxygen species (ROS) (Fig. 3e and f). Oxygen consumption rate (OCR) analysis showed marked reductions in basal and maximal respiratory capacities (Fig. 3g and h), indicating substantial mitochondrial dysfunction. These results establish that the OXA/ART combination therapy induces ferroptotic cell death through the upregulation of iron transport proteins TFRC and DMT1, leading to increased intracellular iron accumulation, enhanced lipid peroxidation, and ultimately mitochondrial dysfunction (Fig. 3i). Expression analysis revealed that combination therapy significantly upregulated the mRNA and protein expressions of the transferrin receptor (TFRC) and divalent metal transporter 1 (DMT1) in Panc-1 and MIA PaCa-2 cells (Fig. 3j, *p* < 0.001) and protein level (Fig. 3k), whereas Western blot analysis revealed that glutathione peroxidase 4 (GPX4) expression levels remained relatively unchanged. In parallel, iron regulatory proteins IRP1 and IRP2 showed no substantial changes in protein abundance, whereas ferritin heavy chain (FTH1) showed a modest increase after ART/OXA treatment (Fig. 3k). These findings suggest that ART/OXA treatment enhances iron uptake-related pathways and induces a compensatory ferritin response, supporting increased intracellular iron availability in association with lipid peroxidation and ferroptotic cell death.

To investigate the upstream regulatory mechanism associated with increased iron-uptake signaling, we examined the nuclear factor erythroid 2–related factor 2 (NRF2) pathway, a key transcriptional regulator of the oxidative stress response and iron metabolism [43–45]. An ARE-luciferase reporter assay revealed that ART/OXA combination treatment significantly increased NRF2 transcriptional activity compared with vehicle or single-agent treatments in both Panc-1 and MIA PaCa-2 cells (Fig. 3l, *p* < 0.01). Consistently, nuclear/cytoplasmic fractionation and immunoblotting demonstrated increased nuclear accumulation of NRF2 following ART/OXA treatment (Sup. Figure 5a). To further evaluate the role of NRF2 in regulating iron-uptake–related genes, siRNA-mediated NRF2 knockdown was performed, resulting in reduced TFRC expression in Panc-1 cells and reduced DMT1 expression in MIA PaCa-2 cells (Sup. Figure 5b). These findings suggest that NRF2 activation contributes to the transcriptional regulation of iron-uptake–related genes in response to ART/OXA treatment. To directly evaluate the functional role of the iron-uptake-related transporters, we performed siRNA-mediated knockdown of TFRC and DMT1 (knockdown efficiencies shown in Sup. Figure 6). Silencing of either TFRC or DMT1 significantly attenuated ART/OXA-induced cytotoxicity (Fig. 3m) and lipid peroxidation (Fig. 3n) in both Panc-1 and MIA PaCa-2 cells, indicating that TFRC- and DMT1-dependent iron uptake contributes to ART/OXA-induced ferroptotic cell death.

### SLC7A11 overexpression mediates dual resistance to oxaliplatin and artesunate

**Fig. 4:**
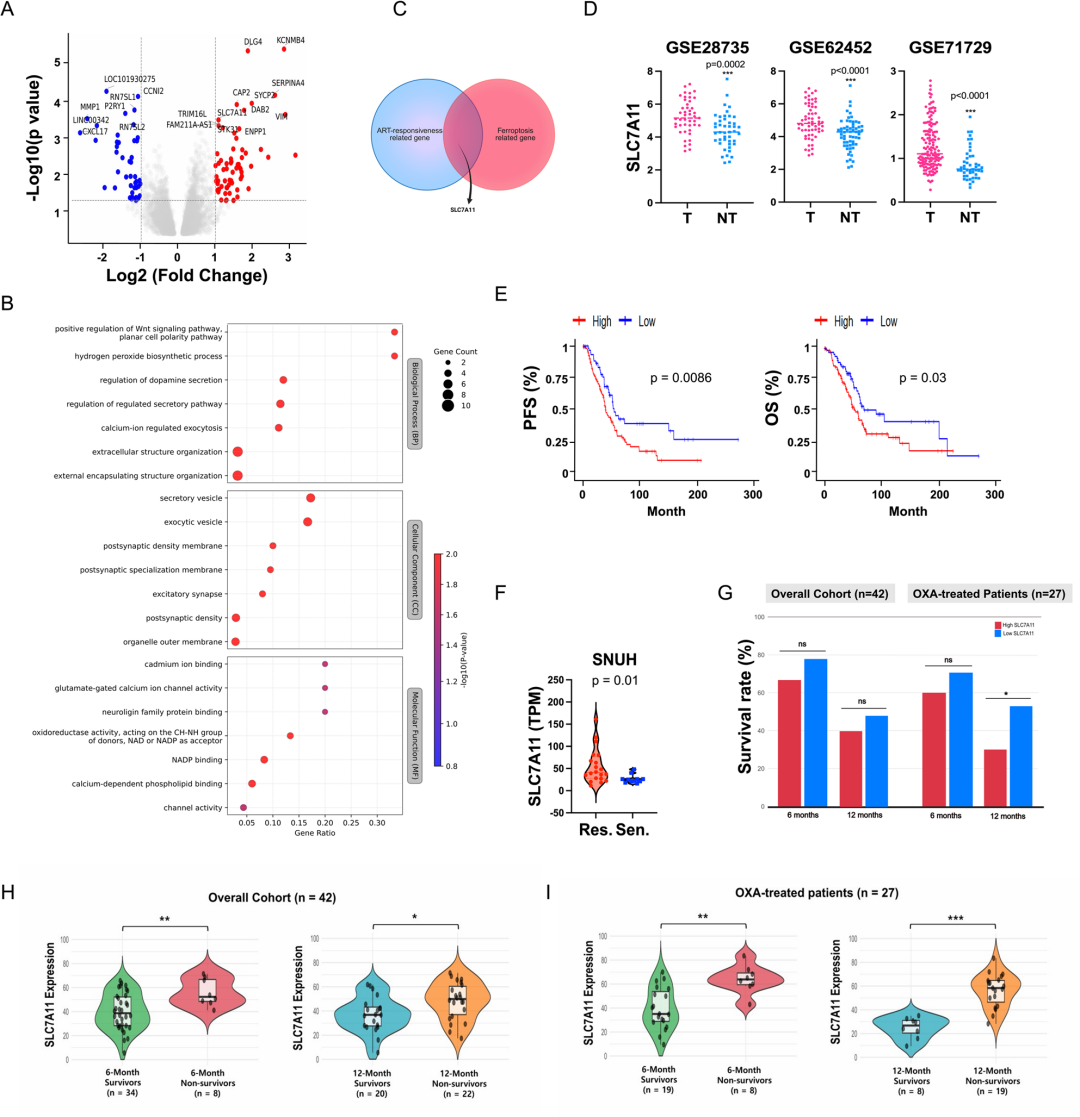
Prognostic impact of SLC7A11 expression in overall and oxaliplatin-treated cohorts. **A** Volcano plot showing differentially expressed genes between dual-resistant and sensitive PDPCOs. Red and blue dots represent significantly up- or downregulated genes, respectively (log2 fold change > 1 or < -1, *p* < 0.05). **B** Gene ontology analysis of differentially expressed genes associated with dual resistance, highlighting enriched processes related to membrane functions and ion binding. **C** Venn diagram showing the intersection between ART-responsiveness-related and ferroptosis-related genes, identifying SLC7A11 as a key overlap. **D** SLC7A11 expression in tumor (T) vs. nontumor tissues across three independent datasets (GSE28735, GSE62452, and GSE71729), with *p*-values indicated. **E** Kaplan–Meier survival curves showing progression-free survival (left panel, *p* = 0.0086) and overall survival (right panel, *p* = 0.03) in pancreatic cancer patients stratified by high (red) vs. low (blue) SLC7A11 expressions. **F** SLC7A11 expression in OXA-resistant vs. OXA-sensitive PDPCOs from the SNUH cohort (*p* = 0.01), shown as TPM. **G** Survival rate comparisons at 6 and 12 months according to SLC7A11 expression level (cutoff: 40) in the overall cohort and oxaliplatin-treated subset. **H** Violin plots comparing SLC7A11 expression levels between survivors vs. non-survivors at 6 and 12 months in the overall cohort (*n* = 42). **I** Violin plots comparing SLC7A11 expression levels between survivors vs. non-survivors at 6 and 12 months in the oxaliplatin-treated subgroup (*n* = 27). Quantitative data are presented as mean ± SEM with individual data points overlaid. Statistical analyses were performed using Student’s t-test for two-group comparisons (**D**, **F**, **H**, **I**) and log-rank test for Kaplan–Meier survival analyses (**E**). *p < 0.05, **p < 0.01, ***p < 0.001; ns, not significant

To investigate mechanisms of acquired dual resistance, we performed transcriptomic comparisons between dual-resistant (OXA/ART) and OXA-only resistant PDPCOs. Transcriptomic analysis revealed distinct gene expression profiles (Fig. 4a), with several upregulated genes in the dual-resistance samples. Gene Ontology analysis showed enrichment in calcium ion binding, channel activity, and membrane-associated processes (Fig. 4b). To identify ferroptosis-related candidates associated with dual resistance, we intersected ART-responsiveness–related genes with ferroptosis-related genes, which identified SLC7A11 as a candidate mediator of acquired dual resistance (Fig. 4c). Public dataset validation confirmed elevated SLC7A11 expression in pancreatic tumors across multiple cohorts (Fig. 4d; GSE28735: *p* = 0.0002; GSE62452: *p* < 0.0001; GSE71729: *p* < 0.0001), with consistently higher expression in tumor (T) versus non-tumor (NT) tissues. High SLC7A11 expression was associated with poorer progression-free survival (Fig. 4e, left panel; *p* = 0.0086) and overall survival (Fig. 4e, right panel; *p* = 0.03) in the TCGA pancreatic adenocarcinoma (PAAD) cohort obtained from the Broad Institute Firehose pipeline [38]. Additionally, SLC7A11 expression was significantly elevated in resistant versus sensitive PC cells (Fig. 4f).

Clinical evaluation in our cohort demonstrated significant prognostic value of SLC7A11. Survival analysis revealed that patients with high SLC7A11 expression (≥ 40) showed numerically lower survival rates at both 6 months (66.7% vs. 77.8%) and 12 months (40.0% vs. 48.1%) compared to those with low expression (< 40) in the overall cohort (*n* = 42), though these differences did not reach statistical significance (*p* = 0.089 and *p* = 0.156, respectively). However, within the oxaliplatin-treated subgroup (*n* = 27), high SLC7A11 expression was significantly associated with worse survival outcomes, with markedly lower 12-month survival rates (30.0% vs. 52.9%, *p* = 0.043) and 6-month differences approaching significance (60.0% vs. 70.6%) (Fig. 4g). When analyzed by survival status, both 6-month and 12-month survivors in the overall cohort demonstrated significantly lower SLC7A11 expression compared to non-survivors (Figs. 4h and 6-month: *p* = 0.006; 12-month: *p* = 0.02). This association was even more pronounced in the oxaliplatin-treated subgroup, where survivors at both time points showed consistently lower SLC7A11 expression (Figs. 4i and 6-month: *p* = 0.001; 12-month: *p* < 0.001). These findings establish SLC7A11 as a candidate biomarker associated with oxaliplatin response and survival in pancreatic cancer.

### SLC7A11 modulation controls ferroptotic sensitivity and overcomes dual resistance

**Fig. 5:**
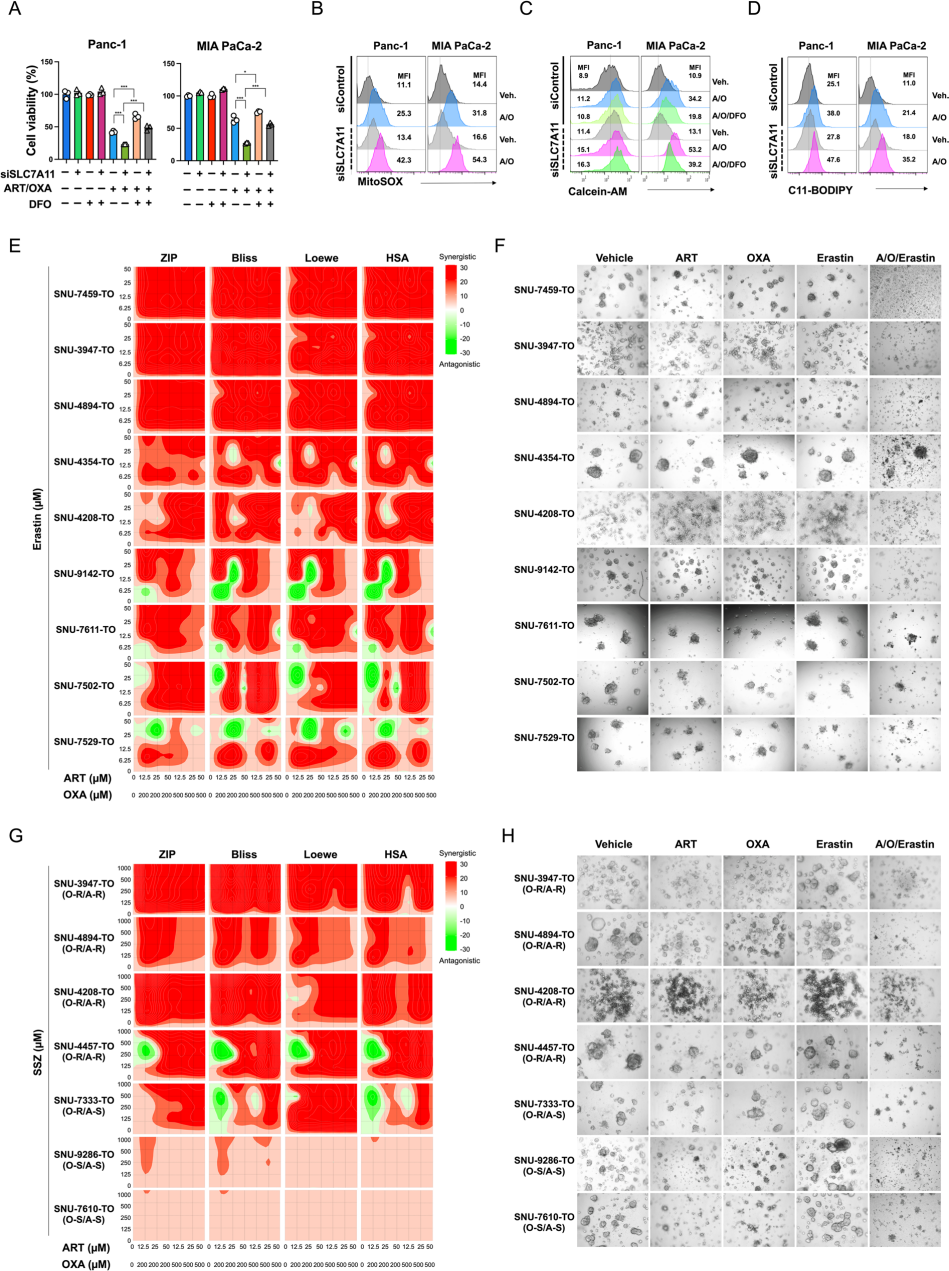
SLC7A11 mediates resistance to oxaliplatin and artesunate via ferroptosis inhibition. **A** Cell viability in Panc-1 and MIA PaCa-2 cells transfected with siControl or siSLC7A11 (20 nM, 48 h), treated with vehicle, ART (20 µM)/OXA (100 µM), and/or DFO (100 µM) for 72 h. **B** Flow cytometric analysis of mitochondrial ROS using MitoSOX (5 µM, 10 min) in siControl or siSLC7A1-transfected cells treated with vehicle or ART (20 µM)/OXA (100 µM) for 24 h. Representative histograms and MFI values shown. **C** Calcein-AM Red (1 µM, 15 min) assay showing labile iron levels in siControl or siSLC7A11 cells treated with vehicle or ART (20 µM)/OXA (200 µM) for 24 h. Higher fluorescence indicates higher iron levels. **D** C11-BODIPY (2 µM, 30 min) staining showing lipid peroxidation in siControl or siSLC7A11 cells treated with vehicle or ART (20 µM)/OXA (200 µM) for 24 h. **E** Synergy maps of erastin (0–50 µM), OXA (200 and 500 µM), and ART (0–50 µM) combinations in PDPCOs using ZIP, Bliss, Loewe, and HSA scoring via SynergyFinder 2.0. The color scale indicates synergistic (red) to antagonistic (green) effects. **F** Representative phase-contrast images of PDPCOs treated with vehicle, ART (20 µM), OXA (200 µM), erastin (10 µM), or ART/OXA/erastin for 4 days. **G** Synergy maps (ZIP, Bliss, Loewe, HSA) of OXA/ART/sulfasalazine triple combination across seven PDPCOs stratified by resistance status (O-R/A-R, O-R/A-S, O-S/A-S). **H** Representative brightfield images of PDPCOs treated with vehicle, OXA, ART, sulfasalazine, or OXA/ART/sulfasalazine triple combination. Quantitative data are presented as mean ± SEM with individual data points overlaid (*n* = 3 independent experiments). Statistical analyses were performed using Student’s t-test. Drug synergy scores in (**E**, **G**) were calculated using SynergyFinder 2.0. *p < 0.05, **p < 0.01, ***p < 0.001; ns, not significant

To functionally validate the role of SLC7A11, we silenced or pharmacologically inhibited SLC7A11 in resistant models. siRNA-mediated SLC7A11 knockdown was confirmed by immunoblotting (Sup. Figure 7a) and significantly reduced cell proliferation (Sup. Figure 7b), migration and invasion (Sup. Figure 7c), spheroid size (Sup. Figure 7d), and spheroid formation capacity (Sup. Figure 7e). siRNA-mediated silencing of SLC7A11 significantly enhanced ART/OXA-induced cytotoxicity in Panc-1 and MIA PaCa-2 cells (Fig. 5a, *p* < 0.001), an effect reversed by deferoxamine treatment, supporting ferroptosis as a resistance-related mechanism. SLC7A11 knockdown also increased mitochondrial ROS production (Fig. 5b), intracellular iron levels (Fig. 5c), and lipid peroxidation (Fig. 5d) in SLC7A11-silenced cells, further supporting its role in ferroptosis suppression.

To complement the loss-of-function data, we generated SLC7A11-overexpressing (SLC7A11-OE) Panc-1 and MIA PaCa-2 cells using a lentiviral expression system, with successful overexpression confirmed at both mRNA and protein levels (Sup. Figure 8a). SLC7A11 overexpression did not affect basal cell proliferation, migration, or invasion (Sup. Figure 8b, 8c), but significantly elevated intracellular GSH levels (Sup. Figure 8d), consistent with enhanced cystine uptake through the System Xc⁻ antiporter. Functionally, SLC7A11-OE cells exhibited increased resistance to both OXA and ART, as reflected by rightward shifts in dose-response curves and substantial IC₅₀ elevations (Sup. Figure 8e). Importantly, ART/OXA-induced cell death was attenuated in SLC7A11-OE cells, with additional protection observed following treatment with the ferroptosis inhibitor Fer-1 (Sup. Figure 8f). Consistently, both lipid peroxidation (Sup. Figure 8 g) and labile iron pool expansion (Sup. Figure 8 h) induced by ART/OXA were partially attenuated in SLC7A11-OE cells and attenuated by Fer-1 or DFO, respectively. Together, these gain-of-function data complement the siRNA silencing results and further support the role of SLC7A11 in modulating ART/OXA sensitivity through ferroptosis-associated mechanisms.

### SLC7A11 inhibition sensitizes resistant PDPCOs to OXA/ART combination therapy

Erastin, a selective SLC7A11 inhibitor, was tested in triple-combination therapy with OXA and ART across nine dual-resistant (OXA resistance, O-R; ART resistance, A-R) PDPCOs. Synergistic interactions were observed across multiple models and synergy metrics (Fig. 5e), with especially high synergy scores in SNU-7459-TO and SNU-3947-TO (ZIP: 61.4 and 73.8; Bliss: 66.0 and 89.8, respectively). Although SNU-4894-TO, SNU-7529-TO, and SNU-9142-TO showed moderate synergy scores (ZIP scores: 44.6, 31.5, and 38.2, respectively), all consistently showed positive synergistic interactions across all metrics. Representative images confirmed that single agents had limited effects on PDPCO growth, whereas the triple combination significantly reduced organoid viability (Fig. 5f). This effect was especially notable in models with high baseline resistance, indicating that SLC7A11 inhibition is a viable strategy to overcome treatment resistance and enhance ferroptosis-driven therapy.

To further evaluate the translational potential of this strategy, we tested sulfasalazine (SSZ), an FDA-approved SLC7A11 in combination with OXA and ART across seven PDPCOs stratified by OXA and ART resistance status (Fig. 5g and h; Sup. Table 3). Although SSZ monotherapy showed minimal cytotoxicity (Sup. Figure 2), the triple combination demonstrated a resistance-dependent synergistic pattern. All four dual-resistant (O-R/A-R) PDPCOs showed significant synergy (ZIP 22.3–96.2; all *p* < 0.0001), whereas dual-sensitive (O-S/A-S) PDPCOs exhibited minimal synergy. In three PDPCOs evaluated with both Erastin and SSZ (SNU-3947-TO, SNU-4894-TO, and SNU-4208-TO), synergistic effects were reproduced with both inhibitors, supporting SLC7A11 inhibition as a shared underlying mechanism. Together, these findings support the translational potential of SLC7A11-targeted combination strategies in OXA-resistant PC.

## Discussion

In this study, we demonstrated that artesunate (ART) enhances anticancer effects and shows synergistic activity with oxaliplatin (OXA) in resistant PC cells by inducing ferroptosis through increased intracellular iron accumulation via the overexpression of iron-import proteins. In PC cells with elevated SLC7A11 expression, which showed reduced response to ART, a triple combination including erastin was effective in overcoming resistance. Our study provides robust preclinical evidence for ferroptosis induction effectiveness in PDPCOs and xenograft models recapitulating patient tumors.

Several studies demonstrated ferroptosis-induced anticancer effects in PC cell lines [23, 46–49]. We extended these findings using PDPCOs, which better recapitulate tumor characteristics. ART, a well-established ferroptosis inducer, demonstrated effective tumor cell death and synergistic effects with OXA in resistant PDPCOs. Although ferroptosis-based treatments can overcome gemcitabine resistance, tumor microenvironment interactions require careful consideration [49–51]. Ferroptosis functions as a double-edged sword—inducing cancer cell death but potentially suppressing antitumor immunity by driving immune cell death [52]. Future research should identify the optimal combinations of ferroptosis regulators and develop targeted delivery strategies to maximize the efficacy while minimizing immunosuppression [42]. The novel GPX4-degrading drug N6F11 shows particular promise by selectively triggering cancer cell ferroptosis while enhancing antitumor immunity, highlighting its potential in PC treatment [53]. 

Few studies exist on biomarkers predicting ferroptosis inducer responsiveness. A previous study demonstrated that ART selectively induces ferroptosis in PDAC cell lines harboring constitutively active KRAS mutations, without affecting normal pancreatic ductal epithelial cells [23]. In our study, we confirmed that ART triggers ferroptosis through iron-dependent mechanisms and identified that high SLC7A11 expression correlates with reduced sensitivity to ART. These findings underscore the potential of SLC7A11 as a predictive biomarker for ferroptosis responsiveness. SLC7A11, the functional subunit of the Xc⁻ system, has been identified as a pan-cancer prognostic biomarker that plays a central role in ferroptosis regulation. Together with ACSL4, GPX4, and SLC3A2, SLC7A11 is among the key molecular determinants of ferroptotic susceptibility in PC [24, 54]. To overcome ART resistance conferred by SLC7A11 expression, we evaluated a combination strategy using erastin, a known SLC7A11 inhibitor. Cotreatment with erastin effectively restored ferroptotic sensitivity in resistant PDPCOs, supporting the efficacy of a dual-targeted therapeutic strategy. This dual stimulation strategy holds potential for clinical translation, but further work is required to optimize delivery and long-term efficacy.

Despite growing studies on ferroptosis-related gene signatures in PC, clinical validation remains lacking. To date, no ferroptosis-targeting agents have been approved for clinical use in PC. The ongoing SPEAR trial, which investigates sulfasalazine, a weak SLC7A11 inhibitor, as a ferroptosis inducer, represents an early step toward clinical evaluation (ACTRN12621001347853). However, the limited potency of sulfasalazine may restrict the interpretability of clinical trial outcomes.

Our findings provide compelling rationale for clinical trials testing ferroptosis inducers like ART added to existing regimens. Stratifying patients by SLC7A11 expression could enhance treatment precision. Additionally, our identification of iron transport proteins, TFRC and DMT1, as key mediators of ART-induced ferroptosis suggests that therapeutic modulation of iron metabolism may offer alternative strategies for overcoming oxaliplatin resistance in PC.

Several study limitations should be acknowledged. Although PDPCO models closely recapitulate the molecular landscape of primary tumors, they lack the complex components of the tumor microenvironment, including stromal and immune elements, that may influence ferroptosis dynamics. To partially mitigate this limitation, we validated our findings in PDPCO-derived xenograft models. Nevertheless, further studies in immunocompetent mouse models are essential to elucidate the interactions between ferroptosis induction and antitumor immunity. While our multiassay characterization of the ferroptotic mechanisms provided robust mechanistic insights, future studies incorporating metabolomics profiling analyses of lipid peroxidation products would further clarify the precise molecular pathways governing ferroptosis. These limitations also highlight important directions for future research: defining optimal drug combinations, developing targeted delivery systems, and tailoring ferroptosis-based therapies to individual molecular profiles for clinical implementation.

Our findings support ferroptosis induction as a novel strategy to sensitize chemoresistant PC. Given the feasibility of repurposing artesunate and ongoing clinical evaluation of SLC7A11 inhibitors, our study provides a compelling rationale for early-phase clinical translation of ferroptosis-targeting strategies in OXA-resistant PC.

## Conclusions

Our study demonstrates that ART overcomes OXA resistance in PC through induction of ferroptosis, mediated by iron transport proteins TFRC and DMT1. We identified SLC7A11 as a critical regulator of resistance to OXA and ART, supporting its potential role as a candidate biomarker of ferroptosis-based therapeutic responsiveness. Importantly, combined treatment with ART, OXA, and SLC7A11 inhibitors resensitized resistant PDPCOs. These findings provide preclinical evidence supporting investigation of ferroptosis-inducing strategies for OXA-resistant PC.

## Supplementary Information

Supplementary Material 1.

Supplementary Material 2.

## Data Availability

The datasets generated and analyzed during the current study are publicly available. Raw RNA-seq and WGS/WES FASTQ files have been deposited in the NCBI Sequence Read Archive (SRA) under the accession number PRJNA1283565. Processed RNA-seq data, including normalized expression matrices and relevant metadata, are available in the Gene Expression Omnibus (GEO) under accession number GEO301526.

## Abbreviations

PC: pancreatic cancer
PDPCO(s): patient-derived pancreatic cancer organoid(s)
OXA: oxaliplatin
ART: artesunate
TFRC: transferrin receptor
DMT1 (SLC11A2): divalent metal transporter 1
SLC7A11: solute carrier family 7 member 11
SLC3A2: solute carrier family 3 member 2
GPX4: glutathione peroxidase 4
NCOA4: nuclear receptor coactivator 4
FTH: ferritin heavy chain
ROS: reactive oxygen species
OCR: oxygen consumption rate
DFO: deferoxamine
Fer-1: ferrostatin-1
z-VAD.fmk: benzyloxycarbonyl-Val-Ala-Asp(OMe)-fluoromethylketone
qPCR: quantitative polymerase chain reaction
DEG(s): differentially expressed gene(s)
GSEA: gene set enrichment analysis
PCA: principal component analysis
NES: normalized enrichment score
FDR: false discovery rate
ZIP: zero interaction potency
HSA: highest single agent
WES: whole-exome sequencing
WGS: whole-genome sequencing
RNA-seq: RNA sequencing
GEO: Gene Expression Omnibus
SRA: Sequence Read Archive
TCGA: The Cancer Genome Atlas
EGF: epidermal growth factor
FGF10: fibroblast growth factor 10
DMEM/F12: Dulbecco’s Modified Eagle Medium/Nutrient Mixture F-12
PBS: phosphate-buffered saline
i.p.: intraperitoneal
siRNA: small interfering RNA
TPM: transcripts per million
MFI: mean fluorescence intensity
H2DCFDA: 2′,7′-dichlorodihydrofluorescein diacetate
C11-BODIPY: lipid peroxidation reporter dye (4,4-difluoro-5,7-dimethyl-4-bora-3a,4a-diaza-s-indacene-C11)
MitoSOX: mitochondrial superoxide indicator
ACSL4: acyl-CoA synthetase long-chain family member 4
AUC: area under the curve
IC50: half maximal inhibitory concentration
