# Supplementary material for: Ferroptosis-based therapeutic strategy targeting iron metabolism and SLC7A11 overcomes oxaliplatin resistance in patient-derived pancreatic cancer organoids
Source: J Exp Clin Cancer Res. 2026 May 30;45:169. doi: 10.1186/s13046-026-03745-z (PMC13435938; doi:10.1186/s13046-026-03745-z)
Supplement: Supplementary file 1 [file 13046_2026_3745_MOESM1_ESM.docx]

**Supplemental material**

1. **Supplemental Figure legends**
2. **Supplemental Tables**
3. **Supplementary methods**

**Supplementary Figure legends**

**Supplemental figure 1. Gene Ontology and GSEA analyses of differentially expressed genes between OXA-sensitive and OXA-resistant PDPCOs.** (A) Gene ontology analysis of differentially expressed genes between OXA-sensitive and OXA-resistant PDPCOs across three categories: biological process (top panel), cellular component (middle panel), and molecular function (bottom panel). Dot size represents the number of genes associated with each term, and color intensity indicates statistical significance (rich factor). (B) GSEA plots for glutathione metabolism and selenocompound metabolism pathways, showing differential enrichment between OXA-sensitive and OXA-resistant PDPCOs. Normalized enrichment scores (NES) and FDR values are indicated.

**Supplemental figure 2. Evaluation of ferroptosis inducers in pancreatic cancer cells.** (A) Cell viability assays in AsPC-1, Panc-1, and MIA PaCa-2 cells treated with varying concentrations of ART (0–50 μM), SOR (0–50 μM), or SSZ (0–10 mM) for 72 hours, with or without ferroptosis inhibitors Fer-1 (1 μM), DFO (1–6 μM), and Trolox (100 μM). (B) Comparative effects of ferroptosis inhibitors and the apoptosis inhibitor zVAD.fmk (10 μM) on ART-induced (0–12.5 μM) cell death in pancreatic cancer cells after 72 hours of treatment, confirming ferroptosis as the primary mode of cell death. Data are presented as mean ± SEM (n = 3 independent experiments). Statistical analyses were performed using Student's t-test. *p < 0.05, **p < 0.01, ***p < 0.001; ns, not significant.

**Supplemental figure 3. Effect of OXA/ART combination on cell migration and invasion.** Migration and invasion assays in AsPC-1, Panc-1, and MIA PaCa-2 cells treated with vehicle, ART (20 μM), OXA (100 μM), or ART/OXA combination for 24–48 hours. Representative images of crystal violet-stained cells (left) and quantification of migrated and invaded cells (right) are shown, with statistical significance indicated with statistical significance indicated. Data are presented as mean ± SEM (n = 3 independent experiments). Statistical analyses were performed using Student's t-test. *p < 0.05, **p < 0.01, **p < 0.001..

**Supplemental figure 4. Heterogeneous sensitivity to ART in patient-derived pancreatic cancer organoids.** Dose–response curves illustrating the variable sensitivity to ART (0–100 μM, 5 days) across 43 PDPCOs, assessed using the 3D CellTiter-Glo assay. Data are presented as mean ± SEM (n = 3 technical replicates per organoid)

**Supplementary Figure 5. NRF2 mediates ART/OXA-induced upregulation of iron-uptake genes in pancreatic cancer cells.** (A) Western blot analysis of Keap1 and NRF2 in nuclear and cytosolic fractions of Panc-1 and MIA PaCa-2 cells treated with vehicle, OXA (40 μM), ART (5 μM), or ART/OXA combination for 24 hours. GAPDH and Lamin A/C were used as cytosolic and nuclear loading controls, respectively. Quantification of nuclear NRF2 normalized to Lamin A/C is shown on the right. (B) Relative mRNA expression of NRF2, TFRC, and DMT1 in Panc-1 and MIA PaCa-2 cells transfected with siControl or siNRF2 (20 nM, 48 hours), measured by qRT-PCR and normalized to β-actin. Data are presented as mean ± SEM (n = 3 independent experiments). *p < 0.05, ***p < 0.001 (Student's t-test).

**Supplementary Figure 6. Validation of siRNA-mediated knockdown of DMT1 and TFRC. (A**) Relative mRNA expression of DMT1 (left) and TFRC (right) in Panc-1 and MIA PaCa-2 cells transfected with siControl, siDMT1, or siTFRC (20 nM, 48 hours), measured by qRT-PCR and normalized to GAPDH. **(B)** Western blot analysis of DMT1 (left) and TFRC (right) in Panc-1 and MIA PaCa-2 cells transfected with siControl, siDMT1, or siTFRC under the same conditions. GAPDH was used as a loading control. Data are presented as mean ± SEM (n = 3 independent experiments). *p < 0.05, **p < 0.01 (Student's t-test).

**Supplemental Figure 7. SLC7A11 knockdown impairs pancreatic cancer cell aggressiveness.** (A) Western blot analysis of SLC7A11 in Panc-1 and MIA PaCa-2 cells transfected with siControl or siSLC7A11 (20 nM, 48 hours). GAPDH was used as a loading control. (B) Cell proliferation measured over 3 days in AsPC-1 and Panc-1 cells transfected with siControl or siSLC7A11 (20 nM), using the CellTiter-Glo assay. (C) Migration and invasion capabilities of Panc-1 cells 48 hours after transfection with siControl or siSLC7A11. Representative images (left) and quantification (right) show remarkably reduced cell migration and invasion following SLC7A11 knockdown. (D) Representative phase-contrast images (left) and spheroid diameter quantification (right) in AsPC-1 and Panc-1 cells transfected with siControl or siSLC7A11 after 5 days in 3D culture. (E) Quantification of spheroid number in the same cells after 5 days in 3D culture, demonstrating remarkable reduction in sphere-forming ability following SLC7A11 knockdown. Data are presented as mean ± SEM with individual data points overlaid (n = 3 independent experiments). Statistical analyses were performed using Student's t-test. *p < 0.05, **p < 0.01, **p < 0.001."

**Supplementary Figure 8. SLC7A11 overexpression confers resistance to ART/OXA-induced ferroptosis in pancreatic cancer cells.** (A) Validation of SLC7A11 overexpression in Panc-1 and MIA PaCa-2 cells transduced with empty lentiviral vector (pLVX) or SLC7A11-expressing construct. Left, relative SLC7A11 mRNA expression quantified by qRT-PCR and normalized to β-actin. Right, Western blot analysis of SLC7A11 protein with GAPDH as loading control. (B) Cell proliferation curves of pLVX and SLC7A11-OE Panc-1 and MIA PaCa-2 cells measured over 4 days by CellTiter-Glo (left). Representative images of 3D spheroid formation are shown on the right. (C) Representative images (left) and quantification (right) of migration (Mig.) and invasion (Inv.) assays in pLVX and SLC7A11-OE cells. (D) Relative intracellular reduced glutathione (GSH) levels in pLVX and SLC7A11-OE cells, measured by GSH-Glo assay. (E) Dose–response curves of OXA and ART in pLVX and SLC7A11-OE Panc-1 and MIA PaCa-2 cells. Corresponding IC₅₀ values are summarized in the table on the right. (F) Cell viability of pLVX and SLC7A11-OE cells treated with vehicle, ART (20 μM)/OXA (200 μM), or ART/OXA + Fer-1 (1 μM, 2-hour preincubation) for 48 hours, assessed by CellTiter-Glo. (G) Relative lipid peroxidation quantified by C11-BODIPY fluorescence under the same treatment conditions as in (F). (H) Flow cytometric analysis of labile iron pool by Calcein-AM staining in pLVX and SLC7A11-OE cells treated with vehicle, ART/OXA, or ART/OXA + DFO (100 μM). Mean fluorescence intensity (MFI) values are indicated in each histogram. Data are presented as mean ± SEM (n = 3 independent experiments). *p < 0.05, **p < 0.01, ***p < 0.001; n.s., not significant (Student's t-test).

**Supplementary Tables**

**Table S1. Characteristics of patients-derived pancreatic cancer organoids.**

| **PDPCO** | **Subtype** | **Age** | **Sex** | **Disease status** | **OXA responsiveness** | **ART responsiveness** |
| --- | --- | --- | --- | --- | --- | --- |
| SNU-3947-TO | Adenocarcinoma | 64 | F | Metastatic | R | R |
| SNU-3969-TO | Adenocarcinoma | 77 | M | Locally advanced | R | S |
| SNU-4208-TO | Adenocarcinoma | 45 | M | Metastatic | R | R |
| SNU-4305-TO | Adenocarcinoma | 59 | F | Locally advanced | R | S |
| SNU-4340-TO | Adenocarcinoma | 66 | M | Metastatic | R | S |
| SNU-4354-TO | Adenocarcinoma | 64 | F | Locally advanced | R | R |
| SNU-4365-TO | Adenocarcinoma | 55 | F | Metastatic | R | S |
| SNU-4378-TO | Adenocarcinoma | 69 | F | Metastatic | R | S |
| SNU-4425-TO | Adenocarcinoma | 60 | F | Locally advanced | R | R |
| SNU-4457-TO | Adenocarcinoma | 58 | M | Metastatic | R | R |
| SNU-4607-TO | Adenocarcinoma | 57 | F | Metastatic | R | R |
| SNU-4837-TO | Adenocarcinoma | 67 | M | Locally advanced | R | R |
| SNU-4894-TO | Adenocarcinoma | 61 | M | Metastatic | R | R |
| SNU-5790-TO | Adenocarcinoma | 45 | F | Metastatic | R | R |
| SNU-5791-TO | Adenocarcinoma | 66 | F | Metastatic | R | S |
| SNU-5813-TO | Adenocarcinoma | 64 | F | Metastatic | R | R |
| SNU-5955-TO | Adenocarcinoma arising from intraductal papillary mucinous neoplasm | 75 | M | Resectable | R | S |
| SNU-6134-TO | Adenocarcinoma | 78 | F | Metastatic | S | S |
| SNU-6390-TO | Adenocarcinoma | 70 | M | Borderline resectable | R | R |
| SNU-6476-TO | Adenocarcinoma | 58 | F | Metastatic | R | S |
| SNU-6831-TO | Adenocarcinoma | 77 | F | Metastatic | R | R |
| SNU-6948-TO | Adenocarcinoma | 70 | F | Metastatic | S | S |
| SNU-6949-TO | Adenocarcinoma | 65 | F | Metastatic | S | S |
| SNU-6992-TO | Adenocarcinoma | 78 | M | Metastatic | S | R |
| SNU-7093-TO | Adenocarcinoma | 77 | M | Metastatic | S | R |
| SNU-7333-TO | Adenocarcinoma | 77 | F | Metastatic | R | S |
| SNU-7388-TO | Adenocarcinoma | 52 | F | Metastatic | S | S |
| SNU-7459-TO | Adenocarcinoma | 72 | F | Borderline resectable | R | R |
| SNU-7502-TO | Adenocarcinoma | 76 | F | Metastatic | R | R |
| SNU-7518-TO | Adenocarcinoma | 73 | M | Metastatic | R | S |
| SNU-7529-TO | Adenocarcinoma | 76 | F | Metastatic | R | R |
| SNU-7610-TO | Adenocarcinoma | 63 | M | Borderline resectable | S | S |
| SNU-7611-TO | Adenocarcinoma | 61 | F | Metastatic | R | R |
| SNU-7634-TO | Adenocarcinoma | 62 | M | Metastatic | R | R |
| SNU-8206-TO | Adenocarcinoma | 60 | F | Metastatic | S | S |
| SNU-8340-TO | Adenocarcinoma | 56 | M | Metastatic | S | S |
| SNU-8341-TO | Adenocarcinoma | 60 | F | Metastatic | R | S |
| SNU-8581-TO | Adenocarcinoma | 71 | M | Metastatic | R | R |
| SNU-9142-TO | Adenocarcinoma | 69 | F | Resectable | R | R |
| SNU-9286-TO | Adenocarcinoma | 76 | F | Borderline resectable | S | R |
| SNU-9381-TO | Adenocarcinoma arising from intraductal papillary mucinous neoplasm | 57 | F | Metastatic | S | R |
| SNU-205A-TO | Adenocarcinoma | 68 | M | Metastatic | R | R |

**Table S2. Synergy scores for ART and OXA combination in patients-derived pancreatic cancer organoids**

| **Synergy Scores Across Models** | | | | | | | | |
| --- | --- | --- | --- | --- | --- | --- | --- | --- |
| PDPCO | ZIP | *p*-value | Bliss | *p*-value | HSA | *p*-value | Loewe | *p*-value |
| SNU-7333-TO | 33.4 | 0.003 | 35.2 | 0.001 | 25.4 | 0.006 | 23.1 | 0.004 |
| SNU-4457-TO | 44.9 | <0.0001 | 44.7 | 0.001 | 21.4 | 0.005 | 20.2 | 0.006 |
| SNU-9286-TO | 56.0 | <0.0001 | 56.8 | <0.0001 | 33.3 | <0.0001 | 44.6 | <0.0001 |
| SNU-4305-TO | 16.7 | <0.0001 | 18.0 | <0.0001 | 21.5 | <0.0001 | 19.3 | <0.0001 |
| SNU-3947-TO | 25.8 | <0.0001 | 25.6 | <0.0001 | 22.0 | <0.0001 | 21.9 | <0.0001 |
| SNU-3969-TO | 44.1 | <0.0001 | 51.3 | <0.0001 | 31.9 | <0.0001 | 29.3 | <0.0001 |
| SNU-7610-TO | 27.5 | <0.0001 | 27.5 | <0.0001 | 34.4 | <0.0001 | 27.2 | <0.0001 |

**Table S3. Synergy scores for OXA/ART/SLC7A11 inhibitor triple combinations across PDPCOs.**

| **Synergy Scores Across Models** | | | | | | | | |
| --- | --- | --- | --- | --- | --- | --- | --- | --- |
| **Erastin + OXA + ART (dual-resistant PDPCO)** | | | | | | | | |
| PDPCO | ZIP | *p*-value | Bliss | *p*-value | HSA | *p*-value | Loewe | *p*-value |
| SNU-7459-TO | 61.4 | <0.0001 | 66.0 | <0.0001 | 65.5 | <0.0001 | 53.8 | <0.0001 |
| SNU-3947-TO | 73.8 | <0.0001 | 89.8 | <0.0001 | 62.4 | <0.0001 | 50.1 | <0.0001 |
| SNU-4894-TO | 44.6 | <0.0001 | 49.1 | <0.0001 | 48.2 | <0.0001 | 47.1 | <0.0001 |
| SNU-4354-TO | 21.8 | <0.0001 | 22.8 | <0.0001 | 26.9 | <0.0001 | 26.8 | <0.0001 |
| SNU-4208-TO | 31.5 | <0.0001 | 34.1 | <0.0001 | 32.9 | <0.0001 | 24.1 | <0.0001 |
| SNU-9142-TO | 18.4 | <0.0001 | 15.7 | 0.02 | 12.7 | 0.05 | 12.7 | 0.03 |
| SNU-7611-TO | 22.0 | <0.0001 | 21.4 | <0.0001 | 22.5 | <0.0001 | 22.6 | <0.0001 |
| SNU-7502-TO | 38.2 | <0.0001 | 44.4 | <0.0001 | 21.3 | <0.0001 | 21.3 | 0.01 |
| SNU-7529-TO | 7.0 | 0.01 | 7.5 | 0.08 | 10.5 | 0.02 | 10.0 | 0.03 |
| **Sulfasalazine (SSZ) + OXA + ART (stratified by status)** | | | | | | | | |
| PDPCO | ZIP | *p*-value | Bliss | *p*-value | HSA | *p*-value | Loewe | *p*-value |
| SNU-3947-TO | 96.2 | <0.0001 | 96.1 | <0.0001 | 42.1 | <0.0001 | 42.3 | <0.0001 |
| SNU-4894-TO | 22.3 | <0.0001 | 23.0 | <0.0001 | 17.3 | <0.0001 | 17.4 | <0.0001 |
| SNU-4208-TO | 66.1 | <0.0001 | 68.9 | <0.0001 | 21.7 | <0.0001 | 40.1 | <0.0001 |
| SNU-4457-TO | 42.6 | <0.0001 | 42.9 | <0.0001 | 32.8 | <0.0001 | 34.6 | <0.0001 |
| SNU-7333-TO | 24.7 | <0.0001 | 11.8 | 0.003 | 85.0 | <0.0001 | 8.0 | 0.02 |
| SNU-9286-TO | 7.9 | <0.0001 | 7.9 | <0.0001 | 4.1 | <0.0001 | 5.1 | <0.0001 |
| SNU-7610-TO | 3.9 | <0.0001 | 3.7 | <0.0001 | 2.3 | <0.0001 | 4.4 | <0.0001 |

**Supplementary methods**

*Cell cultures and chemicals*

Panc-1, MiaPaCa-2, and AsPC-1 cell lines were purchased from the American Type Culture Collection (ATCC; Manassas, VA, USA) and the Korea Cell Line Bank (KCLB, Korea). The AsPC-1 cells were cultured in RPMI (Gibco, CA, USA) containing 10% fetal bovine serum (FBS; Invitrogen, Carlsbad, CA, USA) and 1% penicillin/streptomycin (PS; Gibco). Panc-1, MiaPaCa-2, and BxPC-3 cells were cultured in DMEM (Gibco) containing 10% FBS and 1% PS. Cells were maintained at 37 °C in a humidified atmosphere of 95% air and 5% CO2 and periodically screened for Mycoplasma contamination. Artesunate (ART) was purchased from Tokyo Chemical Company (TCI; Shanghai, China). Oxaliplatin (OXA) was purchased from Sigma-Aldrich (St. Louis, MO, USA). Ferrostatin-1 (Fer-1), Trolox, deferoxamine (DFO), and erastin were purchased from Selleckchem (Houston, TX, USA). Carbobenzoxy-valyl-alanyl-aspartyl-[O-methyl]-fluoromethyl ketone (zVAD.fmk) was purchased from R&D Systems, Inc. (Minneapolis, MN, USA). Sulfasalazine (SSZ) was purchased from Selleckchem. Polybrene and puromycin were purchased from Sigma-Aldrich (St. Louis, MO, USA). Lipofectamine 3000, Lipofectamine RNAiMAX, and Opti-MEM were purchased from Invitrogen (Carlsbad, CA, USA). NE-PER Nuclear and Cytoplasmic Extraction Reagents were purchased from Thermo Fisher Scientific (Rockford, IL, USA).

*Sequencing data processing*

Preprocessing steps included adapter trimming, duplicate marking, base quality score recalibration, and alignment quality control. To identify high-confidence somatic mutations, a multi-step filtering strategy was applied. Initially, only variants with a PASS filter status were retained. Variants were further filtered based on sequencing depth and allele frequency, retaining those with a minimum depth of 25 and a variant allele frequency of at least 0.05. Only bi-allelic single nucleotide variants (SNVs) with unambiguous reference and alternate alleles were included. Variants located in non-coding or synonymous regions, such as intronic, untranslated regions, or intergenic regions, were excluded. The remaining set consisted of protein-altering mutations, including missense variants, frameshifts, splice site alterations, and other functional categories as defined by SnpEff. To remove likely germline variants and common polymorphisms, allele frequency data from the Genome Aggregation Database (gnomAD v4.1)[1] were used. Variants with a East Asian population frequency greater than 1% in either dataset were excluded. Finally, to focus on mutations with established biological or clinical relevance in cancer, the filtered variants were further restricted to those occurring in genes curated in the OncoKB cancer gene database.[2]

For RNA-seq analysis, detailed functional enrichment analysis was conducted using the GSEApy Python package, including KEGG pathway and Gene Ontology term enrichment to identify molecular pathways associated with drug resistance and sensitivity in PDPCOs.[3] Lower AUC values were used to define the sensitive group, while higher AUC values defined the resistant group for OXA and ART cohorts respectively.

***siRNA transfection***

Predesigned siRNAs (AccuTarget™ Genome-wide Predesigned siRNA, SKU SDO-1001) targeting human SLC7A11 (siRNA No. **23657**), TFRC (siRNA No. **7037**), SLC11A2/DMT1 (siRNA No. **4891**), NFE2L2/NRF2 (siRNA No. **4780**), along with **AccuTarget™ Negative Control siRNA (SN-1002)**, were purchased from Bioneer (Daejeon, Korea). Cells were transfected with siRNA (10 or 20 nM final concentration) using Lipofectamine RNAiMAX (Invitrogen) in Opti-MEM (Gibco) according to the manufacturer's instructions. After 48 hours, knockdown efficiency was confirmed by qRT-PCR and Western blotting prior to downstream functional assays.

***Lentiviral SLC7A11 overexpression***

Human SLC7A11 cDNA was obtained from the Korea Human Gene Bank (Korea Research Institute of Bioscience and Biotechnology, Daejeon, Korea) and subcloned into the pLVX-IRES-Puro lentiviral vector (Clontech/Takara Bio, Mountain View, CA, USA). Cloning, sequence verification, and lentiviral packaging quality control were performed by Cosmogenetech (Seoul, Korea). Lentiviral particles were produced in HEK293T cells by co-transfection with packaging plasmids (psPAX2 and pMD2.G) using Lipofectamine 3000. Viral supernatants were collected at 48 and 72 hours post-transfection, filtered through a 0.45 μm filter, and used to transduce Panc-1 and MIA PaCa-2 cells in the presence of polybrene (8 μg/mL). Stably transduced cells were selected with puromycin (2 μg/mL) for 7 days. Empty pLVX-IRES-Puro vector–transduced cells were used as controls. SLC7A11 overexpression was validated by qRT-PCR and Western blotting before downstream experiments.

*qPCR*

For qPCR, total RNA was isolated from cells using the AccuPrep® Universal RNA Extraction Kit (Bioneer, Daejeon, Korea) according to the manufacturer's protocol. Genomic DNA was removed by DNase treatment using RNase-Free-DNase Set (Qiagen, Hilden, Germany). cDNA was synthesized using AccuPower® RocketScript Cycle RT PreMix (Bioneer). Quantitative PCR was performed using AccuPower® GreenStar™ qPCR Master Mix (Bioneer) on an Applied Biosystems 7500 Real-Time PCR System (Thermo Fisher Scientific). Predesigned qPCR primers (AccuTarget™ Human Real-Time PCR Primer, SKU PHS-P02) for GPX4 (Primer No. P130267), SLC7A11 (Primer No. P226802), TFRC (Primer No. P304167), SLC11A2/DMT1 (Primer No. P307447), and NFE2L2/NRF2 (Primer No. P164742) were purchased from Bioneer (Daejeon, Korea). Data were normalized to GAPDH or β-actin expression, with the following primers (5'→3'): GAPDH, (forward) GAAGGTGAAGGTCGGAGTC, (reverse) GAAGATGGTGATGGGATTTC; β-actin (ACTB), (forward) TTCCTGGGCATGGAGTCCTG, (reverse) CGCCTAGAAGCATTTGCGGT. Data analysis was based on the relative quantitative method, and the ΔΔCT value was used to determine the relative fold change in expression.

*Western blotting*

For Western blotting, cells were collected and homogenized in RIPA lysis buffer (Thermo Fisher Scientific) on ice. Subsequently, the cell lysates were centrifuged at 4°C to separate the proteins. Proteins were quantified using a Bicinchoninic Acid (BCA) Protein Assay kit (Thermo Fisher Scientific). Equal amounts of protein were resolved by SDS-PAGE and transferred to PVDF membranes. Membranes were blocked with 5% non-fat milk or BSA in TBS-T for 1 hour at room temperature, incubated overnight at 4°C with primary antibodies, followed by HRP-conjugated secondary antibodies for 1 hour at room temperature, and signals were detected using ECL substrate (Thermo Fisher Scientific). Primary antibodies used in this study were: anti-SLC7A11, anti-DMT1 (SLC11A2), anti-FTH1, anti-GPX4, anti-NRF2, anti-Keap1, anti-IRP1, anti-IRP2, and anti-Lamin A/C (Cell Signaling Technology, Danvers, MA, USA); anti-Transferrin Receptor (Thermo Fisher Scientific); and anti-β-actin and anti-GAPDH (BD Biosciences) as loading controls.

***Subcellular fractionation for nuclear NRF2 detection***

Nuclear and cytoplasmic protein fractions were separated using NE-PER Nuclear and Cytoplasmic Extraction Reagents (Thermo Fisher Scientific) according to the manufacturer's protocol. Briefly, cells treated with vehicle, ART (5 μM), OXA (40 μM), or ART/OXA combination for 24 hours were collected, washed with cold PBS, and lysed sequentially in cytoplasmic extraction reagents followed by nuclear extraction reagent on ice. Equal amounts of nuclear and cytoplasmic proteins were resolved by SDS-PAGE and immunoblotted for NRF2. Lamin A/C and GAPDH were used as nuclear and cytoplasmic loading controls, respectively.

***ARE-luciferase reporter assay***

NRF2 transcriptional activity was measured using a dual-luciferase reporter system. Panc-1 and MIA PaCa-2 cells were seeded in 24-well plates (5 × 10⁴ cells/well) and co-transfected with the ARE-firefly luciferase reporter plasmid (BPS Bioscience, San Diego, CA, USA) and a Renilla luciferase control vector using Lipofectamine 3000 according to the manufacturer's instructions. Twenty-four hours after transfection, cells were treated with vehicle, ART (5 μM), OXA (40 μM), or ART/OXA combination for 24 hours. Luciferase activities were measured using the Dual-Glo Luciferase Assay System (E2920; Promega, Madison, WI, USA) on a GloMax luminometer (Promega). Firefly luciferase activity was normalized to Renilla luciferase activity, and relative ARE activity was expressed as fold change over the vehicle control.

*Drug cytotoxicity in 3D spheroid cultures*

Drug cytotoxicity in 3D spheroid assembly cultures was performed as previously described.[4] Briefly, cells (1,000 cells/well) were seeded in U-bottom-shaped 96-well plates (Corning) and grown into spheroids for 2 days, followed by drug treatment at the above concentrations.

*Drug combination analysis*

Drug combination profiling data were analyzed using SynergyFinder (version 2.0) implementing four reference synergy models and their extensions to calculate synergy scores for higher-order combination data.[5]

*Migration and invasion assay*

Transwell migration assays were performed in a chamber system, and 2–5 × 10^5^ cells suspended in 100 µL DMEM without serum were added to the upper chamber of a 6.5 mm (8 μm pore size) 24-well transwell (Corning).[6] DMEM with 10% FBS was placed into the bottom wells. Invasion assays were the same chamber system using Matrigel (corning) coating. Then, the upper chamber was washed with PBS, fixed with 4% paraformaldehyde for 10 min, and stained with crystal violet. Images were acquired using an inverted microscope (Leica DMi8, Leica Microsystems, Wetzlar, Germany) and calculated staining cells

*Spheres formation assay*

Cells were collected and washed to remove serum, then suspended in serum-free DMEM/F12 supplemented with 20 ng/ml human EGF (PeproTech), 20 ng/ml human recombinant basic fibroblast growth factor (bFGF, PeproTech), and 1XB27 supplement (life technology). The cells were subsequently cultured in ultra-low attachment 96-well plates (Corning) at a density of 2,000 cells/well. Sphere-forming efficacy was calculated by counting the number of spheroids with a size of 100 μm or more.[7]

*ROS detection*

The intracellular ROS and mitochondrial-generated superoxide were determined by H2DCFDA (Invitrogen) and MitoSOX red (Invitrogen) using flow cytometry analysis. Cells were incubated with 10 μM DCFH-DA probe (15 min) and 2.5 μM MitoSox red (30 min) at 37°C. The cell suspension was immediately analyzed on a BD FACSCaliber (BD Biosciences, Franklin Lakes, NJ, USA). All data were processed using the FlowJo™ 10 software.

*Fluorescence microscopy for mitochondrial ROS*

For fluorescence microscopy of mitochondrial ROS, cells were seeded on glass coverslips in 24-well plates and treated with vehicle, ART (20 μM), OXA (200 μM), or ART/OXA combination for 24 hours. After treatment, cells were washed with PBS and incubated with 5 μM MitoSOX Red (Invitrogen) for 15 minutes at 37°C in the dark. Cells were then washed three times with PBS, fixed with 4% paraformaldehyde for 15 minutes, and mounted with DAPI-containing mounting medium (Vector Laboratories, Burlingame, CA, USA). Fluorescence images were acquired using a confocal laser scanning microscope (LSM 800, Carl Zeiss, Germany) with excitation/emission wavelengths of 510/580 nm for MitoSOX Red and 358/461 nm for Hoechst33342. Images were analyzed using Leica Application Suite X (LAS X) software.

***Glutathione (GSH) measurement***

Intracellular reduced glutathione levels were measured using the GSH-Glo Glutathione Assay (V6911; Promega) according to the manufacturer's instructions. Cells (1 × 10⁴/well) were seeded in white-walled 96-well plates and allowed to attach overnight. Following the indicated treatments, GSH-Glo reagent was added directly to the wells and incubated at room temperature for 30 minutes, followed by addition of Luciferin Detection Reagent for 15 minutes. Luminescence was measured on a GloMax luminometer (Promega), and GSH concentrations were calculated using a standard curve.

*Lipid peroxidation assay*

A lipid peroxidation assay was conducted to evaluate for excessive phospholipid peroxidative level, which distinguishes ferroptosis from other cell death mechanisms.[8] The relative malondialdehyde (MDA) concentration in cell lysates was assessed using a Lipid Peroxidation Assay Kit (Abcam, Cambridge, MA, USA) according to the manufacturer’s instructions. Briefly, the MDA in the sample reacted with thiobarbituric acid (TBA) to generate an MDA-TBA adduct. The MDA-TBA adduct was quantified colorimetrically (OD = 532 nm) or fluorometrically (Ex/Em = 532/553 nm).

The relative lipid ROS level in cells was assessed using C11-BODIPY dye (Thermo Fisher Scientific). Cells were treated with 5 μM C11-BODIPY for 30 min, harvested, and washed twice with PBS. Oxidation of the polyunsaturated butadienyl portion of the dye results in a shift of the fluorescence emission peak from ∼590 to ∼510 nm, which was detected by flow cytometry (BD Bioscience). The relative lipid peroxidation level was calculated using the mean fluorescence intensity (MFI).

*Labile iron pool (LIP) assay*

Total cellular iron was measured using the Calcein-AM assay Kit (Thermo Fisher). In brief, cells were washed with PBS and subsequently stained with Calcein-AM (1 μM) in PBS for 15 min at 37°C. Calcein-AM was removed from the cells, and the cell suspension was immediately analyzed on a BD FACSCaliber (BD Biosciences, Franklin Lakes, NJ, USA).[9] Data were processed using the FlowJo™ 10 software.

*Oxygen consumption rate (OCR) measurement*

Mitochondrial respiration was measured using the Seahorse XF Cell Mito Stress Test Kit (Agilent Technologies, Santa Clara, CA, USA) on a Seahorse XFe96 Analyzer according to the manufacturer's instructions. Briefly, cells were seeded in a Seahorse XF96 cell culture microplate at a density of 10,000 cells per well and treated with vehicle, ART (20 μM), OXA (200 μM), or their combination for 24 hours. On the day of the assay, cells were incubated in Seahorse XF DMEM medium supplemented with 10 mM glucose, 1 mM sodium pyruvate, and 2 mM L-glutamine for 1 hour. OCR was measured under basal conditions and after sequential injections of oligomycin (1 μM), FCCP (1 μM), and rotenone/antimycin A (0.5 μM each).

**References**

1.	Chen S, et al. A genomic mutational constraint map using variation in 76,156 human genomes. Nature. 2024; 625: 92-100.

2.	Chakravarty D, et al. OncoKB: A Precision Oncology Knowledge Base. JCO Precis Oncol. 2017; 2017.

3.	Cingolani P, et al. A program for annotating and predicting the effects of single nucleotide polymorphisms, SnpEff: SNPs in the genome of Drosophila melanogaster strain w1118; iso-2; iso-3. Fly (Austin). 2012; 6: 80-92.

4.	Mittler F, et al. High-Content Monitoring of Drug Effects in a 3D Spheroid Model. Front Oncol. 2017; 7: 293.

5.	Ianevski A, Giri AK, Aittokallio T. SynergyFinder 2.0: visual analytics of multi-drug combination synergies. Nucleic Acids Res. 2020; 48: W488-w93.

6.	Pijuan J, et al. In vitro Cell Migration, Invasion, and Adhesion Assays: From Cell Imaging to Data Analysis. Front Cell Dev Biol. 2019; 7: 107.

7.	Kauffman ME, et al. MitoSOX-Based Flow Cytometry for Detecting Mitochondrial ROS. React Oxyg Species (Apex). 2016; 2: 361-70.

8.	DeHart DN, et al. Opening of voltage dependent anion channels promotes reactive oxygen species generation, mitochondrial dysfunction and cell death in cancer cells. Biochem Pharmacol. 2018; 148: 155-62.

9.	Miotto G, et al. Insight into the mechanism of ferroptosis inhibition by ferrostatin-1. Redox Biol. 2020; 28: 101328.
